# *Lactococcus lactis *M4, a potential host for the expression of heterologous proteins

**DOI:** 10.1186/1475-2859-10-28

**Published:** 2011-04-26

**Authors:** Nanyan Noreen, Wei Yeng Hooi, Ali Baradaran, Mohamad Rosfarizan, Chin Chin Sieo, Md Illias Rosli, Khatijah Yusoff, Abdul Rahim Raha

**Affiliations:** 1 Department of Cell and Molecular Biology, Faculty of Biotechnology and Biomolecular Sciences, Universiti Putra Malaysia, 43400 UPM Serdang, Selangor Darul Ehsan, Malaysia; 2 Department of Bioprocess Technology, Faculty of Biotechnology and Biomolecular Sciences, Universiti Putra Malaysia, 43400 UPM Serdang, Selangor Darul Ehsan, Malaysia; 3 Department of Microbiology, Faculty of Biotechnology and Biomolecular Sciences, Universiti Putra Malaysia, 43400 UPM Serdang, Selangor Darul Ehsan, Malaysia; 4 Institute of Bioscience, Universiti Putra Malaysia, 43400 UPM Serdang, Selangor Darul Ehsan, Malaysia; 5 Department of Bioprocess Engineering, Faculty of Chemical Engineering, Universiti Teknologi Malaysia, 81310 UTM Skudai, Johor Darul Takzim, Malaysia

## Abstract

**Background:**

Many plasmid-harbouring strains of *Lactococcus lactis *have been isolated from milk and other sources. Plasmids of *Lactococcus *have been shown to harbour antibiotic resistance genes and those that express some important proteins. The generally regarded as safe (GRAS) status of *L. lactis *also makes it an attractive host for the production of proteins that are beneficial in numerous applications such as the production of biopharmaceutical and nutraceutical. In the present work, strains of *L. lactis *were isolated from cow's milk, plasmids were isolated and characterised and one of the strains was identified as a potential new lactococcal host for the expression of heterologous proteins.

**Results:**

Several bacterial strains were isolated from cow's milk and eight of those were identified as *Lactococcus lactis *by 16S rRNA sequence analysis. Antibiotic susceptibility tests that were carried out showed that 50% of the isolates had almost identical antibiotic resistance patterns compared to the control strains MG1363 and ATCC 11454. Plasmid profiling results indicated the lack of low molecular weight plasmids for strain M4. Competent *L. lactis *M4 and MG1363 were prepared and electrotransformed with several lactococcal plasmids such as pMG36e, pAR1411, pAJ01 and pMG36e-GFP. Plasmid isolation and RE analyses showed the presence of these plasmids in both M4 and the control strain after several generations, indicating the ability of M4 to maintain heterologous plasmids. SDS-PAGE and Western blot analyses also confirmed the presence of GFP, demonstrating the potential of heterologous protein expression in M4.

**Conclusions:**

Based on the 16S rRNA gene molecular analysis, eight Gram-positive cocci milk isolates were identified as *L. lactis *subsp. *lactis*. One of the strains, *L. lactis *M4 was able to maintain transformed low molecular weight plasmid vectors and expressed the GFP gene. This strain has the potential to be developed into a new lactococcal host for the expression of heterologous proteins.

## Background

*Lactococcus lactis *is a lactic acid bacterium possessing the status of generally regarded as safe (GRAS). It is usually used in the dairy and fermented food industry since many decades. It has great potential application in modern biotechnology, which can complement with its long safe history in food fermentation as starter culture. *L. lactis *is currently used as commercial starter cultures in the production of most cultured dairy products and is favourable due to their desirable properties such as rapid acid production that contributes to specific flavours and subtle aromas of fermented food products [1]. Many research and development are being carried out focusing on its applications in the production of biopharmaceuticals and drug delivery.

The genome of several *Lactococcus *strains have been fully sequenced such as *L. lactis *subsp. *lactis *IL1403 [2], *L. lactis *subsp. *cremoris *SK11 [3] and *L. lactis *subsp. *cremoris *MG1363 [4]. The latter strain is by far the most extensively studied bacterium and is widely used as a model for LAB in a wide variety of biotechnological applications. The availability of the full genome sequence of *L. lactis *facilitates the success in annotating complex metabolic pathways in other *L. lactis *strains. For instance, Kleerebezem *et al. *[5] highlighted several examples of metabolic engineering of *L. lactis *for the production of compounds such as diacetyl, alanine and exopolysaccharides, which are important for food industry. These authors reviewed a number of elegant genetic tools used to reroute carbon metabolism in *L. lactis*. On top of that, this bacterium is proved to be an efficient cell factory for the production of food ingredient [6], nutraceuticals [7], heterologous proteins [8,9] and vaccine delivery [10,11].

Due to the efficiency of *L. lactis *as a cell factory, a number of expression systems have been studied, developed and reviewed [12]. One of the most prominent lactococcal expression systems studied and discussed is the nisin-controlled expression (NICE) system [12,13] which is now commercially available. The system works through a signal transduction which occur via two components signal transduction machinery; membrane anchored sensor protein (NisK) and cytoplasmic response regulator protein (NisR). Upon its interaction with nisin, NisK is autophosphorylated and thus, resulting in phosphotransfer to NisR. When NisR is activated, the transcription of the target genes cloned downstream of the nisin promoter P_nisA_, is activated [14]. This system allows tightly regulated protein expression, not only in *Lactococcus*, but also in other LAB such as *Lactobacillus*, *Streptococcus *and *Bacillus *[15,16]. A wide range of successful applications involving this system have been carried out and discussed [14,16,17]. The investigations to date also indicate that different strains of the bacteria differ in their performance due to slight variations in their genetic make up. A number of studies have been conducted to screen and isolate new strains for enhanced performance or to discover novel properties. A host, which can harbour and stably maintain the recombinant plasmids, or strains, which lack certain proteases and thus help in reducing the proteolytic activities and increase the stability of proteins [18], are of interest when studying these new isolates for their specific features. A great deal of interest has also been shown to genetically modify these economically important organisms to improve their traits. Before a new isolate could be used for these studies, the underlying information including its characteristics and genetic make up should be first elucidated. This is important for easier manipulation and control of the strains, as well as fulfilling the requirement of legalisation for its use in food industry.

In the present study, Gram-positive cocci previously isolated from milk were screened for *L. lactis*. The isolates were then identified using 16S rRNA sequencing and some of their genotypic and phenotypic characteristics evaluated. One of the isolates was tested for its ability to harbour and express heterologous plasmids and protein, in order to gauge the potential of the strain to be developed as an expression host.

## Methods

### Bacterial strains and culture condition

The bacterial strains and plasmids used in this study are listed in Table 1. The strains were from a previous laboratory collection isolated from the milk of locally bred cows. The milk was plated aseptically on M17 agar [19] supplemented with 0.5% (w/v) glucose (GM17) and incubated at 30°C for one to two days until colonies appeared. The colonies designated as M2, M4, M5, M6, M11, M12, M14, M16, A1, A2, A3, A4, A5 and A6 were randomly selected for identification.

**Table 1:** Bacterial strains and plasmids

Strain or plasmid	Characteristic	Source or reference
*L. lactis *strains		
M2, M4, M5, M6, M11, M12, M14, M16	Milk isolates	This study
MG1363	*L. lactis *subsp. *cremoris*, plasmid free	[50]
ATCC 11454	*L. lactis *subsp. *lactis*	[25]
*E. coli *strains		
TOP10		Invitrogen
Plasmids		
pMG36e	Em^r^, lactococcal expression vector with constitutive promoter P_32_	[51]
pMG36e-GFP	Em^r^, a derivative of pMG36e carrying GFP gene	Microbial Biotech Laboratory, UPM
pAR1411	Em^r^, lactococcal expression vector	[52]
pAJ01	Em^r^, naturally occuring lactococcal plasmid isolated from chicken cecum	[53]

*L. lactis *subsp. *cremoris *MG1363 and *L. lactis *subsp. *lactis *ATCC 11454 were used as positive controls for the two subspecies of *L. lactis*. All the milk strains and lactococcal controls were cultured in GM17 broth at 30°C for 16 h as stand cultures. *Escherichia coli *TOP10 (Invitrogen) was grown at 37°C in Luria Bertani (LB) broth with vigorous shaking at 200 rpm [20]. Ampicillin, kanamycin and erythromycin were added to final concentrations of 50 μg/ml, 100 μg/ml and 5 μg/ml, respectively. *E. coli *recombinants were screened by the addition of 0.004% (w/v) of 5-bromo-4-chloro-3-indolyl-β-D-galactopyranoside (X-gal) while the *L. lactis *transformants were screened based on the antibiotic resistance selection marker.

### PCR amplification of the 16S rRNA gene fragment

Genomic DNA was extracted using the method described earlier [21]. The partial 16S rRNA gene of the putative lactococcal strains was amplified from their genomic DNA by PCR using primers as described previously [22] with some modifications. The fragment containing the variable regions V1 to V3 of the 16S rRNA gene [23] was amplified using primers P1 (5'-GCG GCG TGC CTA ATA CAT GC-3'; 41-60 [nucleotide number according to the *E. coli *16S rRNA gene]) and P4 (5'-ATC TAC GCA TTT CAC CGC TAC-3'; 685-705) [22]. The PCR products were then run on 1% agarose gel electrophoresis and cloned into pCR2.1-TOPO or pCR-Blunt II-TOPO vectors (Invitrogen).

### Transformation

Transformation of recombinant plasmids into *E. coli *TOP10 strain was done using the heat shock method as described previously [20] with minor modifications. Lactococcal plasmids pMG36e, pAR1411, pAJ01 and pMG36e-GFP were transformed individually into *L. lactis *M4 and MG1363 competent cells by electroporation [24].

### Plasmid profiling

The plasmid profile was generated on agarose gel electrophoresis. The DNA was resolved by 0.7% (w/v) agarose gel and the image was captured after staining with ethidium bromide and UV transillumination. The plasmid sizes were estimated based on the supercoiled plasmids of *L. lactis *ATCC 11454 [25].

### Antibiotic susceptibility test

The antibiotic susceptibility pattern to 16 antibiotics (Table 2) of the *L. lactis *strains was assessed through the agar-disc diffusion method of Kirby-Bauer according to CLSI/NCCLS guidelines (2005) with minor modification [26]. *L. lactis *MG1363 and ATCC 11454 were used as controls. The lactococcal strains were subcultured on GM17 agar plate with sterile cotton swab and were allowed to air-dry. The antibiotic discs (Oxoid) were placed on the agar, four per plate and the cultures were incubated at 30°C overnight and the diameter of the inhibition zone surrounding the antibiotic discs was measured. The test was carried out twice independently and the average of the inhibition zone diameters was calculated.

**Table 2:** Antibiotic discs (Oxoid) used in the antibiotic susceptibility test (source from Flórez *et al.*, 2005)

Antibiotic	Concentration	Mechanism
**Aminoglycoside**		Interfere with protein synthesis
Gentamicin	10 μg	
Kanamycin	30 μg	
Neomycin	30 μg	
Streptomycin	10 μg	
**Coumarin-glycoside**		Interfere with DNA synthesis
Novobiocin	30 μg	
**Penicillin/β-lactam**		Interfere with cell wall synthesis
Ampicillin	10 μg	
Cefamandole	30 μg	
Penicillin G	10 units	
**Macrolide**		Interfere with protein synthesis
Erythromycin	15 μg	
**Peptide**		Interfere with cell wall synthesis
Bacitracin	10 units	
Polymyxin B	300 units	
**Phenicole**		Interfere with protein synthesis
Chloramphenicol	30 μg	
**Quinolone**		Interfere with DNA synthesis
Nalidixic acid	30 μg	
**Tetracycline**		Interfere with protein synthesis
Oxytetracycline	30 μg	
Tetracycline	30 μg	
**Other**		
Nitrofurantoin	300 μg	

### Growth study

An overnight culture of *L. lactis *M4 harbouring pMG36e was diluted (1:100) in GM17 broth. A volume of 1 ml of inoculum was centrifuged to separate the pellets and resuspended with 1 ml of 1× PBS buffer. The optical density at 600 nm (OD_600_) was determined using spectrophotometer (Thermo Scientific, USA). A volume of 100 μl of the culture was used for serial dilution and the cells were spread on GM17 agar. This course was repeated every hour. Growth curve for *L. lactis *M4 carrying pMG36e was constructed and the doubling time was calculated.

### Plasmid stability test

Plasmid stability test was carried out based on the method described previously [27] with some modifications. Based on the data obtained from the growth curve, the cells were maintained at exponential phase for more than 100 generations. A volume of 100 μl of overnight culture of *L. lactis *M4 harbouring pMG36e was inoculated into 100 ml GM17 broth in the absence of antibiotic. The cells were grown until the OD_600 _was at upper log phase, and 100 μl of culture was inoculated into fresh 100 ml GM17 broth. This routine was done successively until the cells reached about 120 generations. The cells were then spread onto GM17 agar for every 20 generations and incubated at 30°C for 16 h. A total of 100 colonies were picked randomly and subcultured on GM17 agar supplemented with erythromycin. The stability of the plasmid was determined based on the ability of the colonies to grow on the agar in the presence of the selective pressure.

### Expression of heterologous protein

The plasmid pMG36e-GFP was used to investigate the expression of the recombinant protein in *L. lactis *M4. A 717 bp DNA fragment encoding for GFP protein was amplified using the primers FGFP (AGAGCTCCGATGAGTAAAGGCGA) and RGFP (CCAAGCTTTTATTTGTATAGTTCATCC) from plasmid *BL21 *(*DE3*) *pLysS *pet 32b(+) GFP (obtained from Microbial Biotech Laboratory, UPM) and digested with *Sac*I and *Hind*III. The purified fragment was then ligated to pMG36e which was also digested with the same enzymes to obtain the expression vector pMG36e-GFP. Approximately 2 μl of ligation mixture was mixed with 40 μl of *L. lactis *competent cells and transferred using electroporation.

An overnight culture of *L. lactis *M4 harbouring pMG36e-GFP was diluted (1:40) in GM17 medium supplemented with erythromycin, grown to an optical density at 600 nm of 0.5 and harvested for protein analysis.

Intracellular and cell-associated proteins were prepared using the method described previously [28]. Samples were run on 12% SDS-PAGE Tris/glycine gels and transferred to polyvinylidene fluoride (PVDF) membrane by using the semi-dry blotting method. Upon usage, the membrane was equilibrated with methanol for few seconds. This was followed by soaking the membrane and three pieces of 3MM Whatman paper in transfer buffer. The transfer of proteins from the SDS-PAGE was accomplished by applying a constant current of 65 mA for at least 1 h depending on the size of protein using Hofer transfer units. Next, the membrane was incubated in 1% blocking solution for overnight at 4°C without shaking. Incubation of the membrane with primary antibody (Anti-GFP Rabbit pAb; Calbiochem) 1:2000 diluted in 0.01% (v/v) Tris-Buffered Saline Tween-20 (TBST) was carried out for 2 h at room temperature with shaking. Following this, the membrane was washed 4 times in 0.01% (v/v) TBST with shaking for 5 min each at room temperature. Secondary antibody (Goat Anti-Rabbit IgG Alkaline phosphatase; Calbiochem) incubation was carried out for 2 h with shaking at room temperature. The antibody used was at a dilution rate of 1:5000 in 0.01% (v/v) TBST. Subsequently, the membrane was washed again as above. Finally, incubation in the detection buffer consisting of 3-bromo-4-chloro-5-indolyl phosphate and nitro blue tetrazolium (BCIP/NBT) (Calbiochem) buffer was carried out with gentle agitation at room temperature for 10-15 min. The progress of the reaction was carefully monitored and when the bands with desired intensity appeared, the membrane was quickly rinsed with sterile water. Finally, the membrane was air-dried and kept until further use.

## Results

### PCR amplification and sequence analysis of 16S rRNA gene fragment

**Figure 1:**
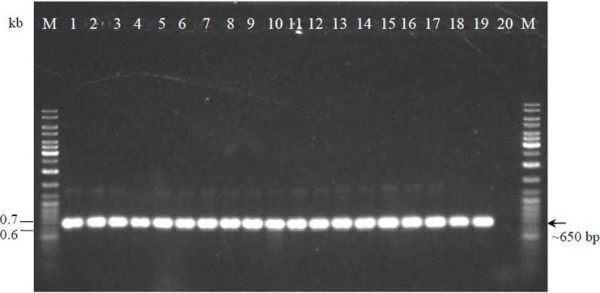
**PCR-amplified partial 16S rRNA gene using P1 and P4 primers**. Lane M: GeneRuler™ DNA Ladder Mix (Fermentas); Lanes 1 to 17: milk isolates; Lane 18: *L. lactis *subsp. *cremoris *MG1363; Lane 19: *L. lactis *subsp. *lactis *ATCC 11454; Lane 20: negative control.

**Figure 2:**
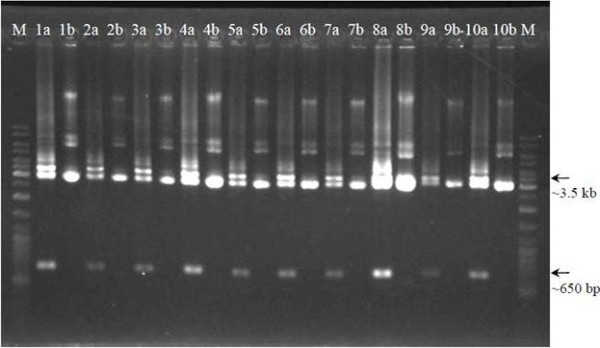
**RE digestion verification of the insertion of partial 16S rRNA gene fragment in the vector**. Lane M: GeneRuler™ DNA Ladder Mix (Fermentas); Lanes 1 to 10: putative recombinant plasmids; Lanes a: *Eco*RI-digestion samples; Lanes b: undigested samples.

The partial 16S rRNA gene fragment was amplified with the primers P1 and P4 (Figure 1) and successfully cloned and verified (Figure 2). These primers targeted the conserved regions of the 16S rRNA gene that flank the variable regions V1 to V3 of the gene [23]. The 90 bp fragment of the V1 region contained sufficient sequence variation to discriminate the species of the genus *Lactococcus *while the V3 region contained sufficient sequence variation to differentiate the species of *Leuconostoc *[22]. Others also reported that the region of greatest heterogeneity occurs in approximately the first 500 bases of the 5'-end [29]. Therefore, the regions of 41-705 nt (*E. coli *numbering) [23] were targeted in this study to identify the milk isolates, instead of the 1.5 kb full length sequence of the gene.

### Plasmid profiling

**Figure 3:**
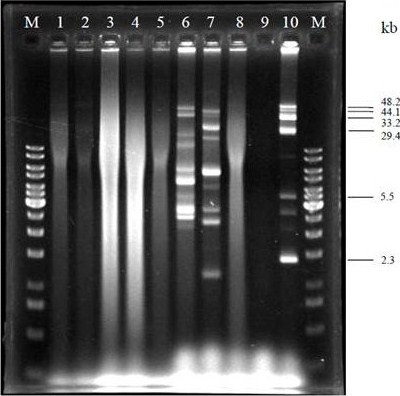
**Plasmid profile of lactococcal strains isolated from raw milk**. Lane M: GeneRuler™ 1 kb DNA Ladder; Lane 1: M2; Lane 2: M4; Lane 3: M5; Lane 4: M6; Lane 5: M11; Lane 6: M12; Lane 7: M14; Lane 8: M16; Lane 9: MG1363; Lane 10: ATCC 11454. The size of plasmids from ATCC 11454 are depicted.

Two of the eight lactococcal strains studied gave plasmid profiles detectable with the method used (Figure 3). These (M12 and M14) lactococcal strains contained several plasmids with various sizes, ranging from 1.6 kb to ~46 kb, and gave different banding patterns that can differentiate them. However, the plasmid extraction of the other six strains was unsuccessful although repeated several times. The plasmid-free strain MG1363 and the nisin-producing strains ATCC 11454 were used as control for the plasmid extraction. The ATCC strain was reported to carry plasmids with the size of 48.2, 44.1, 33.2, 29.4, 5.5 and 2.3 kb [25].

### Antibiotic susceptibility test

The eight lactococcal strains were used for further characterisation studies. Table 3 shows the diameters of the inhibition zone of the antibiotic discs. All of the lactococcal strains exhibited antibiotic susceptibility pattern similar to that of *L. lactis *MG1363 and ATCC 11454. However, some of the strains showed slight differences. Strains M2, M4, M11 and M16 were more resistant to tetracycline (10 μg) and oxytetracycline (30 μg) compared to others.

**Table 3:** Antibiotic susceptibility of *L. lactis *analysed using agar-disc diffusion method

Antibiotic	Diameter of inhibition zone of strains (mm)									
	
	M2	M4	M5	M6	M11	M12	M14	M16	MG1363	ATCC 11454
**Ampicillin**	23	23	22	23	24	26	23	25	21	24
**Bacitracin**	14	12	13	12	13	18	13	14	16	16
**Chloramphenicol**	17	19	16	16	15	19	17	19	18	14
**Gentamycin**	13	13	11	11	12	13	13	12	11	10
**Erythromycin**	20	20	20	18	21	22	19	21	20	21
**Nitrofurantoin**	13	13	11	11	10	11	10	11	13	11
**Kanamycin**	14	13	11	11	13	13	13	12	11	11
**Cefamandole**	19	15	17	18	17	20	17	18	16	20
**Neomycin**	11	11	10	10	12	12	11	11	10	10
**Nalidixic acid**	0	0	0	0	0	0	0	0	0	0
**Novobiocin**	13	14	13	14	12	18	16	13	15	15
**Oxytetracycline**	10	10	21	23	10	23	18	10	24	25
**Penicillin G**	23	20	21	22	23	24	22	22	21	24
**Polymyxin B**	0	0	0	0	0	0	0	0	0	0
**Streptomycin**	8	8	8	7	8	10	9	9	8	9
**Tetracycline**	8	8	20	20	8	20	20	8	23	21

The diameter (in mm) of the inhibition zones were the average of duplicates.

### Verification of *L. lactis *transformants

**Figure 4:**
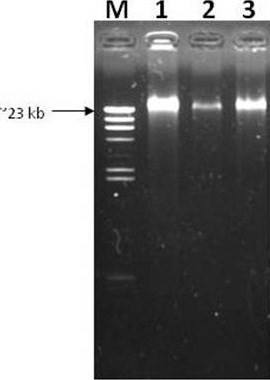
**Total DNA isolation of *L. lactis *subsp. *lactis *M4**. Lane M: λ/*Hind*III marker (Fermentas); Lanes 1 to 3: *L. lactis *M4.

**Figure 5:**
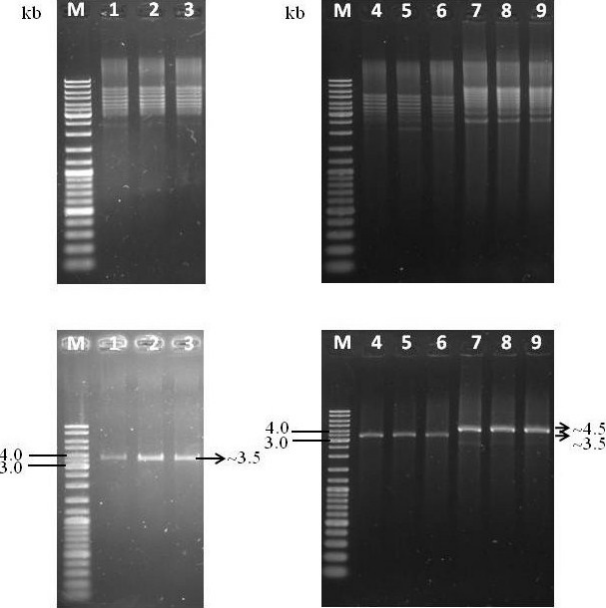
**(Top) Plasmid profile. (Bottom) Verification of transformants by restriction enzyme analysis**. Lane M: GeneRuler™ DNA Ladder Mix (Fermentas); Lane 1: pMG36e in *L. lactis *MG1363; Lanes 2-3: pMG36e in *L. lactis *M4; Lane 4: pAR1411 in *L. lactis *MG1363; Lanes 5-6: pAR1411 in *L. lactis *M4; Lane 7: pAJ01 in *L. lactis *MG1363; Lanes 8-9: pAJ01 in *L. lactis *M4.

**Figure 6:**
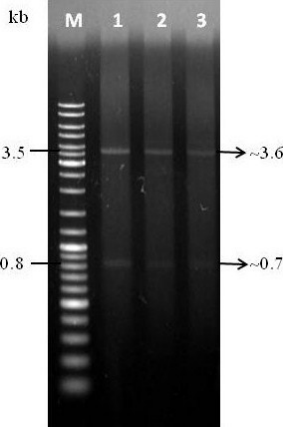
**Verification of *L. lactis *M4 harbouring pMG36e-GFP**. Lane M: GeneRuler™ DNA Ladder Mix (Fermentas); Lanes 1-3: pMG36e-GFP in *L. lactis *M4

Total DNA isolation of *L. lactis *M4 showed that the strain was devoid of low molecular weight plasmid (Figure 4). The strain was transformed with several lactococcal plasmids to investigate its ability to carry plasmid. Based on the plasmid bands extracted from *L. lactis *M4 transformants (Figure 5), it was confirmed that this strain can carry and replicate the plasmids without any indication of possible incompatibility. All plasmids isolated from transformed *L. lactis *M4 cells were shown to have the same sizes as the plasmids isolated from the transformed *L. lactis *MG1363 cells. GFP gene which was cloned into pMG36e was able to be retrieved from digested recombinant plasmid pMG36e-GFP (Figure 6).

### Plasmid stability test

**Figure 7:**
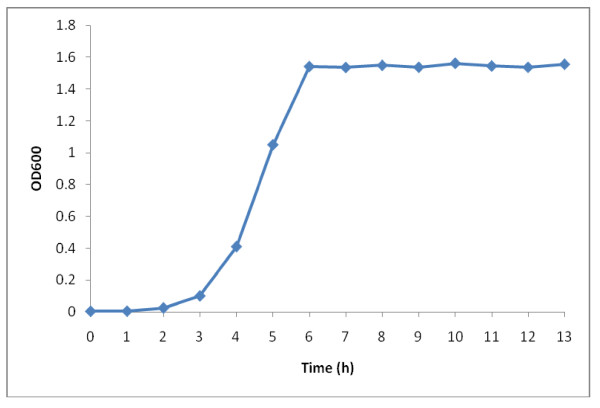
**Growth profile of *L. lactis *M4 carrying pMG36e**.

Based on the growth curve (Figure 7), the doubling time of *L. lactis *M4 carrying pMG36e was found to be 35 min. The doubling time was used to calculate the amount of time needed for the transformant to reach their 100^th ^generations. The stability of the transformed pMG36e in *L. lactis *M4 is crucial to ensure that the plasmid can be maintained in the strain. In this work, all of the 100 colonies were able to grow on the medium containing erythromycin, suggesting that the plasmid is 100% stable in *L. lactis *M4.

### Expression of heterologous protein

**Figure 8:**
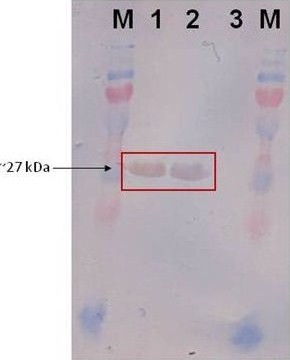
**Western blot analysis**. Lanes M: PageRuler™ Plus Prestained Protein Ladder; Lanes 1-2: pMG36e-GFP clones, Lane 3: Empty *L. lactis *M4 (Negative control)

RE digestion was carried out to confirm the presence of inserts (GFP) by comparing the size differences of the linearised plasmid. Both verifications based on PCR method and double digestion of the recombinant plasmids showed the existence of inserted DNA fragments (Figure 5-bottom). The ability of *L. lactis *M4 and MG1363 to express the recombinant protein GFP was confirmed by SDS-PAGE and Western blot with the presence of a ~27 kDa protein band (Figure 8) (data not shown for the expression of protein in *L. lactis *MG1363).

## Discussion

16S rRNA gene sequence analysis is now widely used as a genetic identification method for bacteria [29]. It is more reliable than the conventional phenotypic method of bacterial identification, which can differentiate even to the species or subspecies level [22,30]. The 16S rRNA gene sequences of the milk strains were compared to the databases available in National Center for Biotechnology Information [NCBI, 31] and Ribosomal Database Project-II [RDP-II, 32] sites. NCBI site hosts and allows access to the general sequence data banks, such as GenBank, European Molecular Biology Laboratory (EMBL) and DNA Data Bank of Japan (DDBJ). However, RDP-II was designed to contain rRNA sequences and provide related services such as sequence alignment and phylogenetic tree building for bacterial identification [33]. Some species within a genus may share identical 16S rRNA gene sequences, for instance, *Mycobacterium bovis *and *M. tuberculosis *[34], therefore 16S rRNA gene sequence analysis of these bacteria may provide limited usage. However, these problems could be solved by polyphasic approach which examines more than one structural gene sequences [29].

The results of this study showed that eight of the 14 milk strains randomly selected from the laboratory collection were identified as *L. lactis *subsp. *lactis*, while the other six strains were found with high sequence similarity with *Enterococcus *species. The 16S rRNA gene sequence analysis (Table 4) showed that only 57% of the randomly selected isolates belonged to the genus *Lactococcus *while the remaining were *Enterococcus*. On the other hand, *L. lactis *subsp. *cremoris *had not been detected in this study. This is not surprising as these microbes are infrequently isolated from raw milk [35]. This was also in accordance with reports that the subsp. *lactis *were more prevalent than *cremoris *in the dairy environment [36]. This may be due to the fact that subsp. *cremoris *occurred in a very small number naturally.

**Table 4:** Sequence similarity of the partial 16S rRNA gene fragment of the milk strains

Strain	Species	Similarity (%)
M2	*Lactococcus lactis *subsp. *lactis*	98
M4	*Lactococcus lactis *subsp. *lactis*	99
M5	*Lactococcus lactis *subsp. *lactis*	100
M6	*Lactococcus lactis *subsp. *lactis*	99
M11	*Lactococcus lactis *subsp. *lactis*	100
M12	*Lactococcus lactis *subsp. *lactis*	99
M14	*Lactococcus lactis *subsp. *lactis*	98
M16	*Lactococcus lactis *subsp. *lactis*	97
A1	*Enterococcus gallinarium*	99
A2	*Enterococcus *sp.	95
A3	*Enterococcus *sp.	98
A4	*Enterococcus *sp.	99
A5	*Enterococcus *sp.	97
A6	*Enterococcus *sp.	98

*L. lactis *was reported to carry multiple plasmids naturally [37]. These plasmids are important for their survival in the natural environment. The dairy lactococci were expected to harbour various plasmids, with different sizes and functions. The plasmid profile of the strains could serve as a typing method to discriminate them [38], and as a source for studying new plasmids. Out of the eight strains of *L. lactis *isolated in this study, only two of them were able to give plasmid profile. Other isolates may contain excessive metabolites such as polysaccharides which retard the extraction method. Extraction method which targeted this type of problem should be tried. Up to nine DNA bands were detected from the plasmid extraction. However, the actual number of plasmids present in the two strains were not elucidated as plasmids may appear in different conformations, such as supercoiled, open circular or linear in the agarose gel analysis. The supercoiled form is normally the most abundant. In addition, some large plasmids may have low copy number. Therefore, they may be difficult to be detected through agarose gel electrophoresis.

Recently, the role of non-pathogenic bacteria in the spread of antibiotic resistance genes, has caused grave concern and is being widely investigated [39,40]. These bacteria have been shown to act as reservoirs for the resistance genes with the potential to transmit them to other microbes. Therefore, in addition to genotypic characterisation, the phenotypic features such as antibiotic resistance of the lactococcal strains were also analysed.

In this study, we report that the strains of *L. lactis *were resistant to antibiotics from the groups of aminoglycosides, coumarin-glycosides and quinolones tested, in addition to the peptide group of polymycin B. These strains were sensitive to the peptide group of bacitracin and macrolides tested. The susceptibilities to β-lactams and phenicole group of antibiotics were in the intermediate range. The resistance of the strains isolated in this study towards aminoglycosides was in agreement with the observation described previously [41]. These antibiotics are not taken up effectively by the bacteria due to their nonoxidative metabolism [42]. 26 strains of *L. lactis *subsp. *cremoris *and subsp. *lactis *were previously reported to be resistant to trimethoporim and sulfathiazole, and the resistance against gentamicin, kanamycin, lincomycin, neomycin, nisin, rifampin and streptomycin varied [41]. Resistance towards tetracyclines varied among the strains isolated in this study, which may due to the acquisition of resistance genes. The results were consistent with the work carried out previously [43], where *L. lactis *was reported to be strongly inhibited by bacitracin but mildly affected by streptomycin. Recently, Flórez *et al. *[44] reported the isolation of several strains of *L. lactis *with high resistance to tetracycline but not to other types of antibiotics.

*L. lactis *M4 exhibited an interesting profile where there was no indication of the presence of low molecular weight plasmids in the total DNA extraction of this strain (Figure 4). When transformed with other lactococcal plasmids, the strain was able to carry them without any signs of incompatibility.

Plasmid incompatibility may become an obstacle in the development of bacterial expression hosts. It can occur when two plasmids failed to be maintained in the same cells [45]. Therefore, the strain M4 was investigated for its ability to carry several lactococcal plasmids in order to check for any incompatibility with low molecular weight plasmids undetected by the plasmid isolation method used in this study. However, incompatibility was not observed as the plasmids were successfully retrieved from the cells (Figure 5).

Several factors have been reported to cause plasmid instability in bacteria including defects in a set of plasmid-coded regulatory elements that regulate replication and partitioning and ineffective synchrony with certain host functions [46,47]. Plasmid instability prevents or decreases the production of desired recombinant proteins carried by some engineered strains, despite their ability to secrete out the proteins to the medium. For example, the use of *Bacillus subtilis *in producing the recombinant proteins has become less favourable because of this [48]. Therefore, the strain M4 was transformed with pMG36e and plasmid stability test was carried out to ensure the ability of the cells to harbour and maintain the plasmid. Based on the growth profile, the cells took about ~60 h to reach 100 generations. Recently, both incubation time and temperature were reported to have substantial effects on plasmid loss [49]. The researchers found that the incubation time had the most extreme effect on the stability of low molecular weight plasmids in *Lactococcus *cells where plasmid loss was observed after 72 h of cultivation while temperatures in the range of 15-40°C also stimulated plasmid instability. Nevertheless, the plasmid pMG36e was found to be 100% stable in *L. lactis *M4 where all of the 100 colonies were able to grow on the medium containing erythromycin even after 120 generations or ~72 h of cultivation.

There is a need to develop suitable microbial hosts for the expression of many important proteins. The current work is important in identifying *L. lactis *M4 as an expression host. Lactic acid bacteria expressing heterologous proteins have been used in the food industry, pharmaceutical and as vaccine delivery. Since they have smaller genome size, do not produce endotoxins, have very low amounts of native exoproteins and are of GRAS status, *Lactococcus *has several advantages over *E. coli *and also *Bacillus sp *as a producer of bioactive molecules. Therefore, the ability of the *L. lactis *M4 to maintain and express the heterologous protein GFP suggest that this strain is potentially ideal to be further characterised and developed as an expression host.

## Conclusions

Our results show that eight *Lactococcus lactis *milk strains were successfully isolated, characterised and were resistant to antibiotics from the groups of aminoglycosides, coumarin-glycosides and quinolones tested, in addition to the peptide group of polymycin B. These strains were also sensitive to the peptide group of bacitracin and macrolides tested. The ability to maintain transformed low molecular weight plasmids and the successful expression of GFP in *L. lactis *M4 indicates that it could be potentially developed as a new lactococcal host for expression of heterologous proteins. Further studies on the characterisation of this strain M4 is needed as this bacterium may carry novel properties that might be useful for various applications beneficial for human use.

## Competing interests

The authors declare that they have no competing interests.

## Authors' contributions

WYH carried out the isolation and characterisation of the isolates. NN carried out the transformation of lactococcal plasmids into the strain M4 and studied their stability. AB performed the expression of GFP in the strain M4. WYH and NN drafted and coordinated the manuscript. ARR, CCS, MR, MIR and KY participated in the discussion of experimental results and in the revision of manuscript's intellectual content. All authors read and approved the final manuscript.

## References

[B1] Joseph FF Ashraf NH Elmer HM, James LS Starter cultures and their use Applied Dairy Microbiology 1998 New York, Marcel Dekker 131 172

[B2] Bolotin A Wincker P Mauger S Jaillon O Malarme K Weissenbach J Ehrlich SD Sorokin A The complete genome sequence of the lactic acid bacterium *Lactococcus lactis *ssp. *lactis *IL1403 Genome Research 2001 11 731 753 10.1101/gr.GR-1697R 11337471 PMC311110

[B3] Makarova K Slesarev A Wolf Y Sorokin A Mirkin B Koonin E Pavlov A Pavlova N Karamychev V Polouchine N Shakhova V Grigoriev I Lou Y Rohksar D Lucas S Huang K Goodstein DM Hawkins T Plengvidhya V Welker D Hughes J Goh Y Benson A Baldwin K Lee JH Díaz-Muñiz I Dosti B Smeianov V Wechter W Barabote R Lorca G Altermann E Barrangou R Ganesan B Xie Y Rawsthorne H Tamir D Parker C Breidt F Broadbent J Hutkins R O'Sullivan D Steele J Unlu G Saier M Klaenhammer T Richardson P Kozyavkin S Weimer B Mills D Comparative genomics of the lactic acid bacteria Proc Natl Acad Sci USA 2006 103 15611 15616 10.1073/pnas.0607117103 17030793 PMC1622870

[B4] Wegmann U O'Connell-Motherway M Zomer A Buist G Shearman C Canchaya C Ventura M Goesmann A Gasson MJ Kuipers OP van Sinderen D Kok J Complete genome sequence of the prototype lactic acid bacterium *Lactococcus lactis *subsp. *cremoris *MG1363 J Bacteriol 2007 189 8 3256 7320 2007 10.1128/JB.01768-06 17307855 PMC1855848

[B5] Kleerebezem M Hols P Hugenholtz J Lactic acid bacteria as a cell factory: rerouting of carbon metabolism in *Lactococcus lactis *by metabolic engineering Enzym Microb Tech 2000 26 840 848 10.1016/S0141-0229(00)00180-0 10862894

[B6] Hugenholtz H The lactic acid bacterium as a cell factory for food ingredient production Int Dairy J 2008 18 466 475 10.1016/j.idairyj.2007.11.015

[B7] Hugenholtz J Sybesma W Groot MN Wisselink W Ladero V Burgess K van Sinderen D Piard JC Eggink G Smid EJ Savoy G Sesma F Jansen T Hols P Kleerebezem M Metabolic engineering of lactic acid bacteria for the production of nutraceuticals Antonie Leeuwenhoek 2002 82 217 235 10.1023/A:1020608304886 12369189

[B8] Le Loir Y Azevedo V Oliveira SC Freitas DA Miyoshi A Bermúdez-Humarán LG Nouaille S Ribeiro LA Leclercq S Gabriel JE Guimaraes VD Oliveira MN Charlier C Gautier M Langella P Protein secretion in *Lactococcus lactis*: an efficient way to increase the overall heterologous protein production Microb Cell Fact 2005 4 2 10.1186/1475-2859-4-2 15631634 PMC545053

[B9] Morello E Bermúdaz-Humarán LG Llull D Sole V Miraglio N Langella P Poquet I *Lactococcus lactis*, an efficient cell factory for recombinant protein production and secretion J Mol Microbiol Biotechnol 2008 14 48 58 10.1159/000106082 17957110

[B10] Bahey-El-Din M Griffin BT Gahan CGM Nisin inducible production of listeriolysin O in *Lactococcus lactis *NZ9000 Microb Cell Fact 2008 7 24 10.1186/1475-2859-7-24 18664263 PMC2515284

[B11] Raha AR Varma NRS Yusoff K Ross E Foo HL Cell surface display system for *Lactococcus lactis*: a novel development for oral vaccine Appl Microbiol Biotechnol 2005 68 75 81 10.1007/s00253-004-1851-8 15635459

[B12] de Vos WM Gene expression systems for lactic acid bacteria Curr Opin Microbiol 1999 2 289 295 10.1016/S1369-5274(99)80050-2 10383867

[B13] de Ruyter PGGA Kuipers OP Beerthuyzen MM van Alen-Boerrigter I de Vos WM Functional analysis of promoters in the nisin gene cluster of *Lactococcus lactis* J Bacteriol 1996 178 3434 3439 8655538 10.1128/jb.178.12.3434-3439.1996 PMC178110

[B14] Mierau I Kleerebezem M 10 years of the nisin-controlled gene expression system (NICE) in *Lactococcus lactis* Appl Microbiol Biotechnol 2005 68 705 717 10.1007/s00253-005-0107-6 16088349

[B15] Pavan S Hols P Delcour J Geoffroy MC Grangette C Kleerebezem M Mercenier A Adaptation of the nisin-controlled expression system in *Lactobacillus plantarum*: a tool to study in vivo biological effects Appl Environ Microbiol 2000 66 4427 4432 10.1128/AEM.66.10.4427-4432.2000 11010894 PMC92320

[B16] Zhou XX Li WF Ma GX Pan YJ The nisin-controlled gene expression system: construction, application and improvements Biotechnology Advances 2006 24 285 295 10.1016/j.biotechadv.2005.11.001 16380225

[B17] Mierau I Olieman K Mond J Smid EJ Optimization of the *Lactococcus lactis *nisin-controlled gene expression system NICE for industrial applications Microb Cell Fact 2005 4 16 10.1186/1475-2859-4-16 PMC1182390 15921537

[B18] Cortes-Perez NG Poquet I Oliveira M Gratadoux JJ Madsen SM Miyoshi A Corthier G Azevedo V Langella P Bermúdez-Humarán LG Construction and characterization of a *Lactococcus lactis *strain deficient in intracellular ClpP and extracellular HtrA proteases Microbiology 2006 152 2611 2618 10.1099/mic.0.28698-0 16946256

[B19] Terzaghi BE Sandine WE Improved medium for lactic streptococci and their bacteriophages J Appl Microbiol 1975 29 807 813 10.1128/am.29.6.807-813.1975 PMC187084 16350018

[B20] Sambrook J Russell DW Molecular cloning: a laboratory manual 2001 third Cold Spring Harbor Laboratory Press

[B21] Leenhouts KJ Kok J Venema G Stability of integrated plasmids in the chromosome of *Lactococcus lactis* Appl Environ Microbiol 1990 56 2726 2735 16348281 10.1128/aem.56.9.2726-2735.1990 PMC184834

[B22] Klijn N Weerkamp AH de Vos WM Identification of mesophilic lactic acid bacteria by using polymerase chain reaction-amplified variable regions of 16S rRNA and specific DNA probes Appl Environ Microbiol 1999 57 3390 3393 10.1128/aem.57.11.3390-3393.1991 PMC183979 1723586

[B23] Neefs JM van de Peer Y Hendriks L de Wachter R Compilation of small ribosomal subunit RNA sequences Nucleic Acids Res 1990 18 Supplement 2237 2317 1692117 10.1093/nar/18.suppl.2237 PMC331875

[B24] Holo H Nes IF High-frequency transformation, by electroporation, of *Lactococcus lactis *subsp. *cremoris *grown with glycine in osmotically stabilized media Appl Environ Microbiol 1989 55 3119 3123 16348073 10.1128/aem.55.12.3119-3123.1989 PMC203233

[B25] Gonzalez CF Kunka BS Transfer of sucrose-fermenting ability and nisin production phenotype among lactic streptococci Appl Environ Microbiol 1985 49 627 633 16346756 10.1128/aem.49.3.627-633.1985 PMC373560

[B26] Kastner S Perreten V Bleuler H Hugenschmidt G Lacroix C Meile L Antibiotic susceptibility patterns and resistance genes of starter cultures and probiotic bacteria used in food Syst Appl Microbiol 2006 29 145 155 10.1016/j.syapm.2005.07.009 16464696

[B27] Imanaka T Aiba S A perspective on the application of genetic engineering: stability of recombinant plasmid Ann New York Acad Sci 1981 369 1 14 10.1111/j.1749-6632.1981.tb14172.x 7020540

[B28] Le Loir Y Gruss A Ehrlich S Langella P A nine-residue synthetic propeptide enhances secretion efficiency of heterologous proteins in *Lactococcus lactis* J Bacteriol 1998 180 7 1895 9537390 10.1128/jb.180.7.1895-1903.1998 PMC107105

[B29] Patel JB 16S rRNA gene sequencing for bacterial pathogen identification in the clinical laboratory Mol Diagn 2001 6 313 321 11774196 10.1054/modi.2001.29158

[B30] Heikens E Fleer A Paauw A Florijn A Fluit AC Comparison of genotypic and phenotypic methods for species-level identification of clinical isolates of coagulase-negative staphylococci J Clin Microbiol 2005 43 2286 2290 10.1128/JCM.43.5.2286-2290.2005 15872257 PMC1153770

[B31] National Center for Biotechnology Information http://www.ncbi.nlm.nih.gov/accessed in January 2008

[B32] Ribosomal Database Project-II http://rdp.cme.msu.edu/accessed in January 2008

[B33] Cole JR Chai B Farris RJ Wang Q Kulam-Syed-Mohideen AS McGarrell DM Bandela AM Cardenas E Garrity GM Tiedje JM The ribosomal database project (RDP-II): introducing *myRDP *space and quality controlled public data Nucleic Acids Res 2007 35 Database D169 D172 10.1093/nar/gkl889 17090583 PMC1669760

[B34] Hall L Doerr KA Wohlfiel SL Roberts GD Evaluation of the MicroSeq system for identification of mycobacteria by 16S ribosomal DNA sequencing and its integration into a routine clinical mycobacteriology laboratory J Clin Microbiol 2003 41 1447 1453 10.1128/JCM.41.4.1447-1453.2003 12682128 PMC153882

[B35] Desmasures N Mangin I Corroler D Guéguen M Characterization of lactococci isolated from milk produced in the Camembert region of Normandy Appl Microbiol 1998 85 999 1005 10.1111/j.1365-2672.1998.tb05264.x 9871320

[B36] Nomura M Kobayashi M Narita T Kimoto-Nira H Okamoto T Phenotypic and molecular characterization of *Lactococcus lactis *from milk and plants J Appl Microbiol 2006 101 396 405 10.1111/j.1365-2672.2006.02949.x 16882147

[B37] McKay LL Functional properties of plasmids in lactic streptococci Antonie van Leeuwenhoek 1983 49 259 274 10.1007/BF00399502 6312881

[B38] Kuhl SA Larsen LD McKay LL Plasmid profiles of lactose-negative and proteinase-deficient mutants of *Streptococcus lactis *C10, ML3, and M18 Appl Environ Microbiol 1979 37 1193 1195 16345399 10.1128/aem.37.6.1193-1195.1979 PMC243377

[B39] Salyers AA Gupta A Wang Y Human intestinal bacteria as reservoirs for antibiotic resistance genes Trends Microbiol 2004 12 412 416 10.1016/j.tim.2004.07.004 15337162

[B40] Wang HH Manuzon M Lehman M Wan K Luo H Wittum TE Yousef A Bakaletz LO Food commensal microbes as a potentially important avenue in transmitting antibiotic resistance genes FEMS Microbiol Lett 2006 254 226 231 10.1111/j.1574-6968.2005.00030.x 16445749

[B41] Orberg PK Sandine WE Survey of antimicrobial resistance in lactic streptococci Appl Environ Microbiol 1985 49 538 542 3922298 10.1128/aem.49.3.538-542.1985 PMC373544

[B42] Foster TJ Plasmid-determined resistance to antimicrobial drugs and toxic metal ions in bacteria Microbiol Rev 1983 47 361 409 6355806 10.1128/mr.47.3.361-409.1983 PMC281581

[B43] Angelotti R Weiser HH Slatter W Gould IA The effect of antibiotics upon the microflora of milk Appl Microbiol 1955 3 234 237 13239116 10.1128/am.3.4.234-237.1955 PMC1057107

[B44] Flórez AB Delgado S Mayo B Antimicrobial susceptibility of lactic acid bacteria isolated from a cheese environment Can J Microbiol 2005 51 51 58 10.1139/w04-114 15782234

[B45] Hashimoto-Gotoh T Inselburg J ColE1 plasmid incompatibility: localization and analysis of mutations affecting incompatibility J Bacteriol 1979 139 608 619 378980 10.1128/jb.139.2.608-619.1979 PMC216910

[B46] Uhlin BE Nordström K Plasmid incompatibility and control of replication: copy mutants of the R-factor R1 in *Escherichia coli *K-12 J Bacteriol 1975 124 641 649 1102525 10.1128/jb.124.2.641-649.1975 PMC235950

[B47] Novick RP Plasmid incompatibility Microbiol Rev 1987 51 381 395 3325793 10.1128/mr.51.4.381-395.1987 PMC373122

[B48] Lynch HC Argyropoulos D Kotsarinis M Chuen-Im S Cell size differences in plasmid-containing and plasmid-free cells of *Bacillus subtilis *during batch and cyclic batch culture Enzym Microb Tech 2000 26 34 39 10.1016/S0141-0229(99)00124-6

[B49] Avşaroğlu MD Buzrul S Şanlıbaba P Alpas H Akçelik M A kinetic study on the plasmid stability of three *Lactococcus lactis *strains J Ind Microbiol Biotechnol 2007 34 729 737 10.1007/s10295-007-0249-x 17726621

[B50] Gasson MJ Plasmid complements of *Streptococcus lactis *NCDO 712 and other lactic streptococci after protoplast-induced curing J Bacteriol 1983 154 1 9 6403500 10.1128/jb.154.1.1-9.1983 PMC217423

[B51] van de Guchte M van de Vossen JMBM Kok J Venema G Construction of a lactococcal expression vector: expression of hen egg white lysozyme in *Lactococcus lactis *subsp. *lactis* Appl Environ Microbiol 1989 55 224 228 2495760 10.1128/aem.55.1.224-228.1989 PMC184082

[B52] Hooi WY Raha AR Mariana NS Construction of an expression vector for *Lactococcus lactis *based on an indigenous cryptic plasmid Afr J Biotechnol 2009 8 21 5621 5626

[B53] Raha AR Ross E Yusoff K Manap MY Ideris A Characterisation and molecular cloning of an erythromycin resistance plasmid of *Lactococcus lactis *isolated from chicken cecum J Biochem Mol Biol Biophys 2002 6 7 1 10.1080/10258140290010151 12186776

